# A putative role for lactate in the crosstalk between chemokines and glycolysis in solid cancer

**DOI:** 10.7150/ijbs.91108

**Published:** 2024-03-29

**Authors:** Selma Rivas-Fuentes, Teresa Santos-Mendoza, Alfonso Salgado-Aguayo

**Affiliations:** 1 Laboratory of Transcriptomics and Molecular Immunology, Instituto Nacional de Enfermedades Respiratorias “Ismael Cosío Villegas”, Mexico City, Mexico; 2 Laboratory of Research on Rheumatic Diseases, Instituto Nacional de Enfermedades Respiratorias “Ismael Cosío Villegas”, Mexico City, Mexico

**Keywords:** cancer, chemokines, glycolysis, lactate

## Abstract

Chemokines are very important for carcinogenesis and the development of a malignant phenotype. Lactate is a small molecule produced during glycolysis; recently it has emerged as an immunomodulator that could impact tumor cell behavior. In this paper we explore the interplay between chemokines, glycolysis, and lactate in cancer progression, and propose the existence of a pro-tumoral lactate-chemokine-glycolysis loop driven by high glucose levels.

Several members of the chemokine family are currently known to be involved in the pathophysiology of cancer, since they induce proliferation, angiogenesis, invasion, metastasis, and the generation of a pro-inflammatory microenvironment 1. On the other hand, a very active glucose metabolism represents a favorable condition for tumor growth and for the reprogramming of the metabolism of tumor cells necessary for its survival. Studies in tumors show that the concentration of lactate (a byproduct of glycolysis), the expression of glucose transporters, and the levels and activity of glycolytic enzymes, are increased 2. Lactate can induce various cellular responses, including cell migration and angiogenesis 2. Although these processes are typical chemokine-induced functional responses and they are necessary for cancer progression, few studies address the role of lactate in the context of chemokine biology in cancer.

Based on the empirical data presented below, we propose that in high extracellular glucose, lactate could be a link between chemokine signaling pathways and glycolysis, and in consequence induce a pro-tumoral loop. In this context, chemokine stimulation may promote the glycolytic pathway activation, and lactate produced by glycolysis could induce chemokine expression (see Figure 1).

**Empirical data:** Under high extracellular glucose, the expression of angiogenic chemokines is positively regulated. In THP-1 cells, a human monocytic cell line, it was demonstrated that high extracellular glucose deregulates the expression of relevant immune related genes. 41 out of 375 analyzed genes were upregulated, including several chemokines such as CCL2, CXCL10, CCL20, CCL17, CCR2B, CCR4, and CCR5, as well as several adhesion molecules 3. Studies show that the expression of CXCR4 in Jurkat cells, a human lymphocytic cell line, and of CCL2 and CX3CL1 in smooth muscle cells, is increased under high glucose 4, 5. Importantly, some chemokines can affect the glycolytic pathway, for example, CXCL10 induces aerobic glycolysis in T lymphocytes through the induction of hexokinase 1 (HK1) and the glucose transporters Glut-1, 2, 3 and 4; interestingly, lactate inhibited HK-1 and decreased its induction by CXCL10, limiting glycolysis in the context of the non-transformed cell. 6 Furthermore, in *in vitro* activated human T cells, CCL5 regulated glucose uptake through sustained expression of GLUT-1, and induced signaling of AMPK, a key molecule in the regulation of glycolysis. 7

The aggressive phenotype of tumor cells might be increased in high extracellular glucose, since chemokines are modulators of proliferation, migration, invasion, and resistance to apoptosis 1. Also, lactate produced by glycolysis could induce chemokine and chemokine receptor expression favoring cancer. For example, exposure to lactic acid promotes murine colorectal cancer progression by generating a metastatic niche in bone by inducing CXCL10 expression in osteoclast precursors 8. Thus, in humans, it is possible that lowering local lactate concentrations may hinder the establishment of metastases. Furthermore, as lactic acidosis has been associated with radioresistance 2, this treatment may be more effective with such an intervention.

Another important consequence of high lactate is the promotion of tumor-associated macrophages (TAMs) with a pro-tumoral phenotype. Paolini *et al*. reported that chronic exposure to lactate has important effects on the differentiation of human monocytes into pro-tumoral TAMs, since they secrete EGF, TGF, IL-20, VEGF-A, IL-1β, IL-6, TNF-α and CXCL8, and at the messenger level, CXCL8, CCL2, CCL13, CXCL5, CXCL2, CXCL3, CXCL1, CCL18 and CCL24 are increased 9. Their experiments co-culturing monocytes with a primary human ovaric cancer cell line showed that exposure to lactic acid polarized macrophages towards a pro-tumoral phenotype, which could facilitate tumor vascularization, tumor growth and later metastasis formation.

In human pituitary adenoma, lactate dehydrogenase enzyme levels, an indicator of lactate production, were found to be increased in invasive compared to non-invasive tumor samples and in larger tumors, which also exhibit a higher presence of TAMs with a protumoral phenotype that facilitate invasion by secretion of CCL17. Medium acidification by the secretion of lactate by neoplastic cells induced the polarization of TAMs to the protumoral M2 phenotype 10.

In addition, lactate could have immunomodulatory functions in activated T cells since lactate inhibits their chemotaxis, thus inhibiting T cell effector functions against tumor cells 6. Interestingly, it has been reported that in CD4^+^ T cells, sodium lactate but not lactic acid blocks glucose uptake and glycolytic efflux 6, which implies that local microenvironments are relevant in this context.

**In conclusion,** high extracellular glucose induces overexpression of some chemokines which upregulate the glycolytic pathway by increasing HK1 activity and the production of lactate. In turn, high lactate levels increase the local expression of chemokines which favor metastasis, promote the generation of protumoral macrophages, and decrease the antitumor immune response, establishing a pro-tumor lactate-chemokine-glycolysis loop. In this context, pharmacological inhibition of this loop, for example by targeting lactate production with specific drugs and/or locally using chemokine neutralizing antibodies or chemokine receptor neutralizing peptides, could positively affect the outcome of the cancer patient.

The crosstalk between chemokines and glycolysis in solid cancer may be especially relevant in diabetic patients coursing with cancer due to the upregulation of proangiogenic chemokines in hyperglycemia, which could contribute to cancer progression. Therefore, it is important to conduct further research on the lactate-chemokine-glycolysis loop in cancer to understand the molecular mechanisms involved in the aforementioned loop, which should consider the pleiotropic functions of chemokines and the ionization state of lactate.

## Figures and Tables

**Figure 1:**
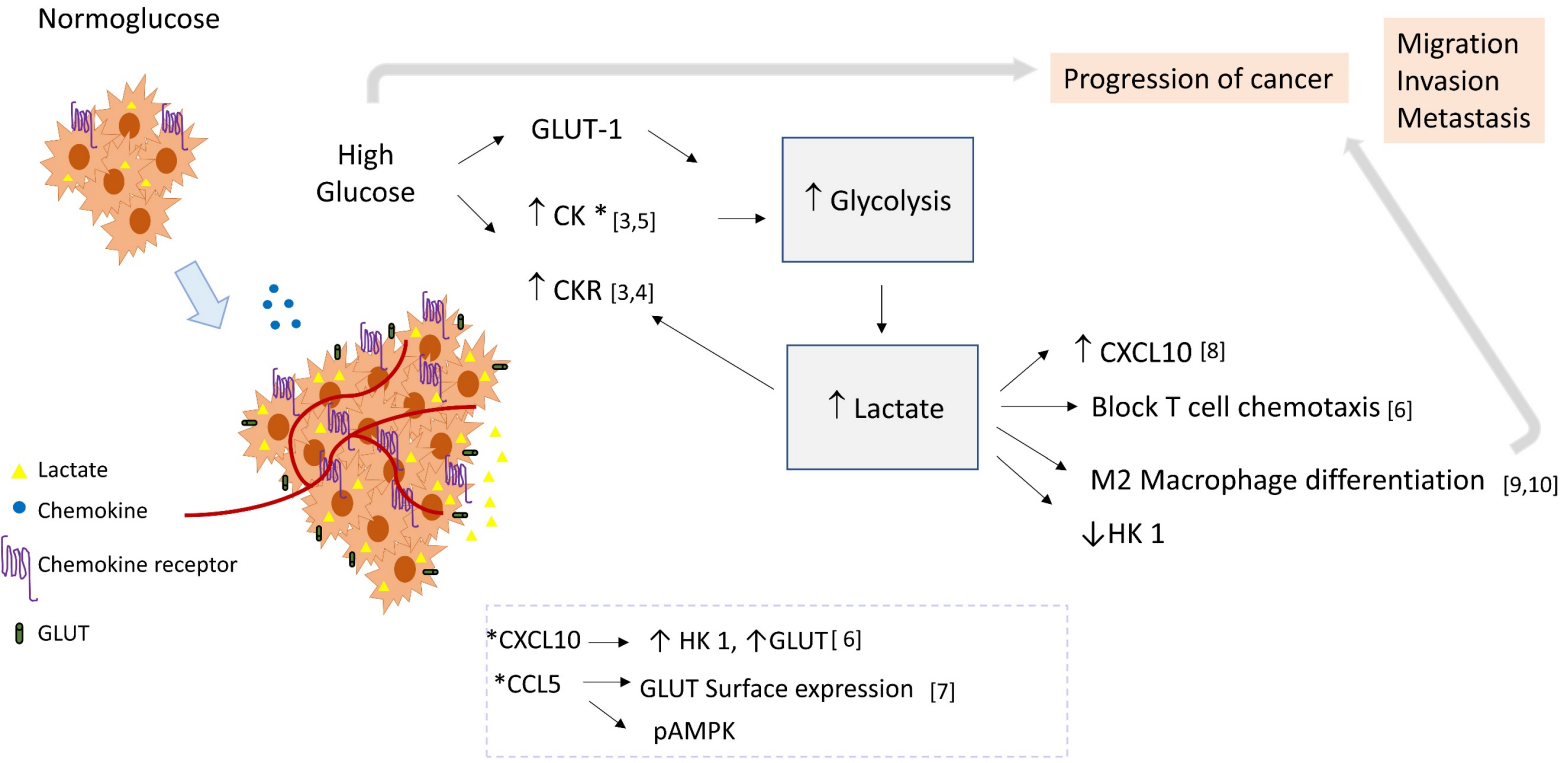
** Role of lactate as a crosstalk molecule between chemokines and glycolysis in cancer.** Cellular activation by chemokines may induce the glycolytic pathway and glycolytic enzymes, thereby contributing to lactate production. Lactate could favor the tumoral environment supporting macrophage differentiation, increasing the expression of chemokines, and blocking T cell chemotaxis. Then lactate could establish a positive feedback loop, favoring inflammation and tumor progression by linking chemokine and glycolysis pathways. Abbreviatures: HK 1 (Hexokinase 1), GLUT-1 (Glucose transporter 1), CK (Chemokine), CKR (Chemokine Receptor).
